# Unsolved Questions in the Revascularization of Older Myocardial Infarction Patients with Multivessel Disease

**DOI:** 10.31083/j.rcm2310344

**Published:** 2022-10-14

**Authors:** Rita Pavasini, Federico Sanguettoli, Luca Zanarelli, Maria Angela Deserio, Nicola Bianchi, Gioele Fabbri, Matteo Tebaldi, Simone Biscaglia, Gianluca Campo

**Affiliations:** ^1^UO Cardiologia, Azienda Ospedaliero Universitaria di Ferrara, 44124 Ferrara, Italy

**Keywords:** coronary artery disease, multivessel CAD, myocardial infarction, elderly, older

## Abstract

**Background:**

In cardiology, the global phenomenon of population ageing poses new major challenges, ranging from more comorbid and frail patients to the presence of complex, calcified and multiple coronary lesions. Considering that elderly patients are under-represented in randomized clinical trials (RCT), the aim of this systematic review is to summarize the current knowledge on the revascularization of the elderly patient with myocardial infarction and multivessel coronary artery disease.

**Methods:**

A systematic review following PRISMA guidelines has been performed. The search was conducted on Pubmed (Medline), Cochrane library, Google Scholar and Biomed Central databases between January and February 2022. We selected the articles focusing on patients hospitalized for myocardial infarction (MI) with multivessel disease and aged 75 years or older. A total of 36 studies have been included.

**Results:**

Multivessel coronary artery disease is present in around 50–60% of older patients with MI. The in-hospital mortality rate of patients older than 75 years is double compared to their younger counterpart, and the most prevalent complications after revascularization are bleeding and renal failure. In the treatment of patients with ST elevation MI (STEMI), primary percutaneous coronary intervention should be the first choice over fibrinolysis. However, it is not clear whether this population would benefit from complete revascularization or not. In patients with non-ST elevation MI (NSTEMI), an invasive approach with either percutaneous coronary intervention or coronary artery bypass graft may be chosen, but a conservative strategy is also accepted. There are no data from large trials about the comparison of possible revascularization strategies in NSTEMI patients.

**Conclusions:**

This systematic review shows that this field of research lacks randomized clinical trials to guide revascularization strategy in older STEMI or NSTEMI patients with MI. New results are expected from ongoing trials.

## 1. Introduction

The aging of the population is a phenomenon that physicians worldwide have to face [1]. Often, due to the presence of multivessel CAD, chronic and highly calcified lesions, older patients are usually frail, comorbid, and with more complex coronary artery disease (CAD) [2]. At the state of the art, there is a lack of data from randomized clinical trials (RCTs) answering the questions on managing coronary revascularization in elderly patients. Most published trials on patients aged 75 years and older are observational, retrospective and dated [3]. This age group of patients tends to be excluded from large clinical trials, where mean age of patients is usually around 65–70 years [4]. Therefore, there is lack of representation of the real world population that usually develops acute coronary syndromes (ACS) and needs revascularization strategies: the octogenarians, the segment of the population that is showing the largest growth in percentage related to the increase in life expectancy [2]. Therefore, the aim of this systematic review is to summarize main evidence related to the management of older patients with myocardial infarction and multivessel coronary artery disease.

## 2. Materials and Methods 

**Fig. 1.**
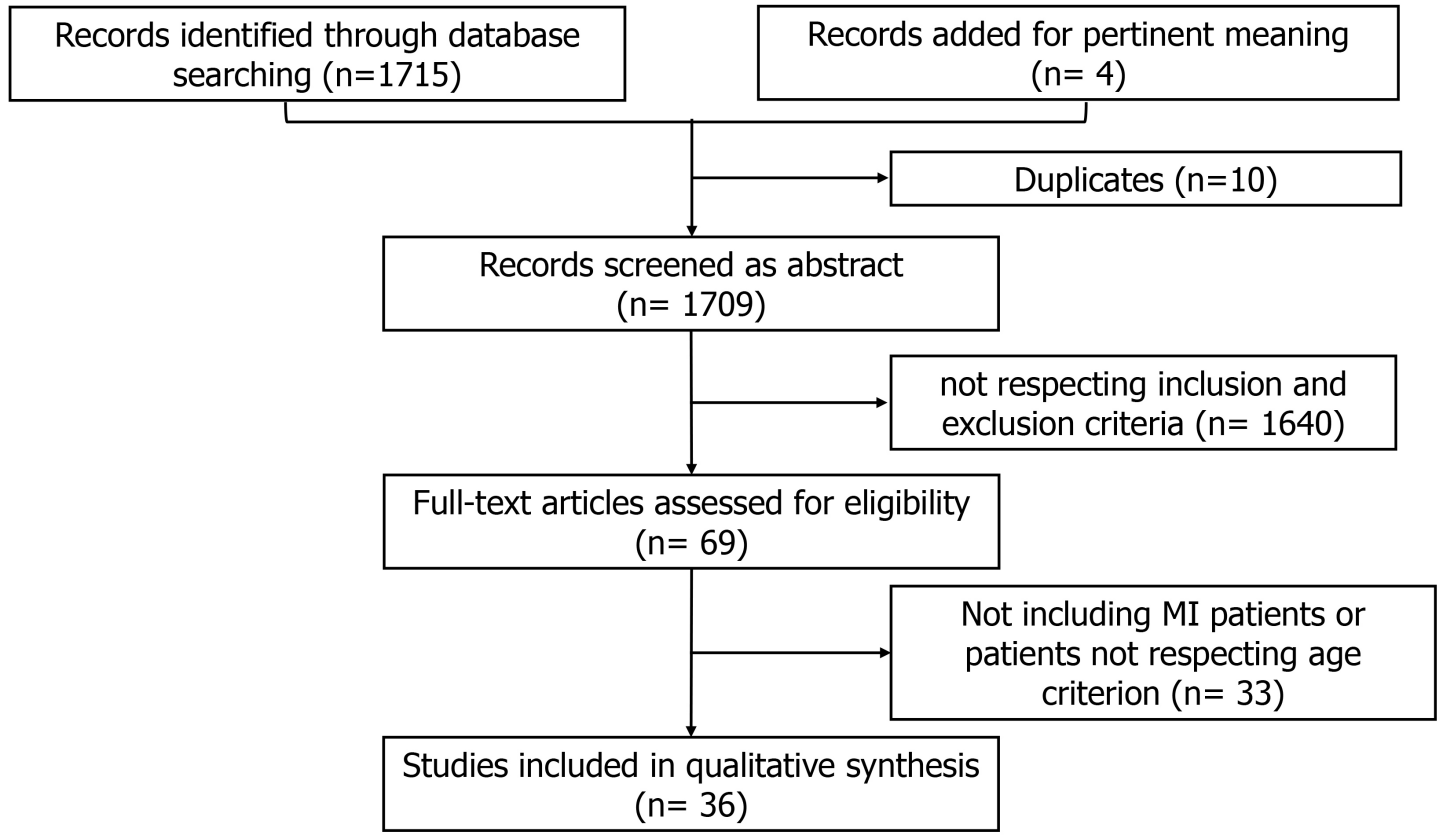
**Search strategy details**. MI, myocardial infarction.

We performed a systematic review following Preferred Reporting Items for Systematic reviews and Meta-Analyses (PRISMA) [5] guidelines. The search was carried out between January and February 2022. Pubmed (Medline), Cochrane library, Google Scholar and Biomed Central have been used as databases. The primary aim of the present systematic review was identifying the studies including (i) patients hospitalized for myocardial infarction with multivessel disease and (ii) aged 75 years or older. The terms searched were: ((PCI) OR (percutaneous coronary intervention)) AND ((multivessel disease) OR (multivessel coronary artery disease) OR (trivessel)) AND ((older) OR (elderly)). The study types we considered were (i) observational trial, (ii) randomized clinical trials, and (iii) meta-analysis. Only papers published in English and in peer review journals have been selected. Overall, 1715 records were found from database search, and four were added after the screening of references of relevant studies. After removing duplicates, we excluded further 1673 articles as they were irrelevant based on their title and abstract (Fig. 1). Finally, we settled on 36 relevant studies. The quality of the included studies was tested using pre-specified electronic forms of MINORS criteria [6]. The minimum score obtained was 13, and the maximum was 16. No more studies were excluded based on quality assessment.

## 3. Results

### 3.1 Prevalence of Multivessel CAD in Elderly Patients

The prevalence of multivessel CAD is higher in older people. This concept has been known since late ‘80s and early ‘90s, a time when most patients were treated only with medical therapy, as this subgroup of patients was considered to be at higher risk of perioperative mortality [7]. In a retrospective study by Reyen *et al*. [7] dated back to 1992, 75% of patients over 75 years of age had multivessel disease and/or involvement of the left main, in comparison to 54% of patients aged less than 75 years. Maiello *et al*. [8] reported almost the same prevalence of multivessel disease in older MI patient (72%), whereas other authors found slightly lower rate, around 60–65% [9, 10]. More recent reports from the European and American registries continue to underline how high multivessel CAD is in elderly patients (aged 75 years and older), with prevalence setting at around 50–60% when a stenosis of more than 70% is found in at least two vessels [11, 12] (Table 1, Ref. [7, 8, 9, 10, 13, 14, 15, 16, 17, 18, 19, 20, 21, 22, 23, 24, 25, 26, 27, 28, 29, 30, 31, 32, 33, 34, 35, 36, 37, 38, 39, 40]).

**Table 1.** **Studies included in the systematic review on patients aged 75–89 years**.

Studies on older patients (75 to 89 years) and multivessel CAD					
References	Study type	N. Older Patients/overall population	Mean Age years	% of multivessel CAD	Outcome
Prevalence of multivessel CAD in elderly patients					
Reyen *et al*. (1997) [7]	Retrospective	398/398	78 ± 3	70%	Age per se is not a contraindication to perform PCI.
Maiello *et al*. (1992) [8]	Retrospective	47/47	77 ± 1.5	72%	Percutaneous coronary angioplasty is a valid therapeutic alternative in elderly patients with CAD.
Buffet *et al*. (1992) [9]	Retrospective	102/102	77	79%	PCI is quite effective in most patients and brings long-term relief of symptoms with an excellent long-term survival.
Thompson *et al*. (1991) [10]	Retrospective	193/752	79	63%	In very elderly patients, coronary angioplasty is usually successful, but extra caution is warranted.
STEMI in the elderly: how to manage revascularization					
De felice *et al*. (2011) [13]	Retrospective	75/524	75	48%	In patients undergoing rescue PCI at 1 year of follow-up the mortality and MACE rates were significantly greater in patients aged >75 years.
Khera S. *et al*. (2013) [14]	Retrospective	90567/356.358	84.3 ± 3.6	not reported	Between 2001 and 2010 a decreasing trend in STEMI, an increasing trend in PCI utilization for STEMI, and reduction in in-hospital mortality were observed.
Chen *et al*. (2010) [15]	Retrospective	76/201	78 ± 2	80%	Complete revascularization of the very old patients might improve the prognosis and reduce the incidence of cardiac events.
de La Torre Hernandez *et al*. (2018) [16]	Registry	1830/3576	81.1 ± 4	100%	Multivessel PCI is related with better two years outcomes, but the benefit seems to be greater for staged procedures.
Rumiz *et al*. (2018) [17]	Prospective observational	111/381	81.5 ± 4	100%	Routine CR strategy in the elderly may not confer a clear clinical benefit during a long-term follow-up; whereas it could be the best option in younger patients.
Joshi *et al*. (2020) [18]	RCT sub-group analysis	110/627	80	37% (3-vessel disease)	In patients ≥75 years, after treatment of the culprit lesion in STEMI, there is no significant prognostic benefit to prophylactic CR of non-culprit stenoses.
Biscaglia *et al*. (2022) [19]	Retrospective	2087/5470	81.5	100%	CR is associated with lower mortality if compared to a culprit-only strategy, with a similar safety profile.
NSTEMI in the elderly: how to manage revascularization					
Dacey LJ *et al*. (2007) [20]	Retrospective	1693/1693	83	100%	Favourable survivorship for octogenarians undergoing either CABG or PCI for treatment of multivessel coronary artery disease.
Sheridan BC *et al*. (2010) [21]	Prospective	10141/10141	87.7	100%	In very elderly patients with ACS and multivessel CAD, CABG appears to offer an advantage over PCI of survival and freedom from composite endpoint at three years.
Sliman H *et al*. (2019) [22]	Retrospective	139/139	84.5 ± 3.6	80%	In the elderly population with left main disease, heart team decision making should be implemented when discussing revascularization options.
Posenau *et al*. (2016) [23]	Retrospective	763/763	79 ± 3	100%	CABG was associated with the best overall clinical outcomes, but was selected for a minority of patients.
Hannan *et al*. (2014) [24]	Retrospective	3864/3864	77	100%	Older patients experienced similar mortality and stroke/MI/mortality rates for CABG and PCI with DES, although repeat revascularization rates were higher for patients undergoing PCI with DES.
Harada *et al*. (2016) [25]	Registry (prospective observational)	322/1923	81.0 ± 3	100%	In elderly patients over 75 years of age, CR-PCI appears to reduce MACE at one year, independent of other risk factors.
Complication after MI in older patients					
Rynkowska-Kidawa M. *et al*. (2015) [26]	Retrospective	82/82	88.6	64%	In octogenarian patients aged 85 years and older, PCI appears to be a reasonably safe and effective procedure, especially in patients with stable coronary disease.
Wanha *et al*. (2016) [27]	Retrospective	1916/1916	75	65%	Elderly patients have increased risk of in-hospital bleeding complications requiring blood transfusion and a higher risk of death at 12-month follow-up. The use of new-generation DES reduces the risk of MI in the elderly population.
Oe K *et al*. (2003) [28]	Retrospective	193/1655	83.4 ± 2.8	59.6%	Impaired myocardial reserve may contribute to a large portion of in-hospital deaths in octogenarians with ACS.
Bromage *et al*. (2016) [29]	Retrospective	1051/1051	84.2	54%	Octogenarians undergoing primary PCI has a higher rate of complications and mortality compared with a younger population.
Zimmerman *et al*. (2006) [30]	Prospective	115/504	80 ± 4	60%	Radial artery access diminishes bleeding complications, particularly in the elderly. In 30-days survivors of STEMI, age and presence of multivessel disease are independent predictors of 1-year mortality.
Cardarelli F *et al*. (2009) [31]	Retrospective	7383/169826	86	67%	Risk stratification for patients with acute MI should incorporate an assessment of renal function with estimated GFR values.
Prognosis of older patients after ACS					
Sakai *et al*. (2002) [32]	Retrospective	261/1063	80.8 ± 4.6	50%	When reperfusion is successful, the cardiac mortality rate in older patients is not significantly higher than in younger patients.
Teplitsky I *et al*. (2003) [33]	Retrospective	97/97	85	65%	Cardiogenic shock has a profound negative prognostic impact on octogenarians despite ‘aggressive’ PCI attempts.
Ipek G *et al*. (2016) [34]	Retrospective	186/2931	83 ± 4	65.9%	Acute stent thrombosis, anterior MI, heart failure, low ejection fraction, ventricular arrhythmias and multivessel disease are the independent risk factors for in-hospital mortality among octogenarian patients after primary PCI.
Renilla *et al* (2013) [35]	Retrospective	102/102	87.5 ± 2.5 years	60%	Mortality and morbidity in very elderly patients with STEMI are very high, especially in those not receiving reperfusion therapies. Heart failure on admission is an independent risk factor for hospital mortality.
Erriquez *et al*. (2021) [36]	Retrospective	586 (586)	78 ± 5	80.5%	In a large cohort of older adults admitted to hospital for NSTEMI undergoing PCI, large periprocedural MI was associated with long-term occurrence of all-cause and cardiovascular mortality.
Campo *et al*. (2019) [37]	Retrospective	402/402	78 ± 6	74%	The assessment of the physical performance with SPPB scale before hospital discharge increases the ability to predict adverse events in older ACS patients.
Caretta G *et al*. (2014) [38]	Retrospective	139/728	85.1 ± 3.9	35%	Older age, LVEF <40% on admission, hemodynamic instability (higher Killip class or low SBP), and post-interventional TIMI flow <3 are independent predictors of mortality.
Yamanaka F *et al*. (2013) [39]	Retrospective	1494/9877	84.4 ± 4	65.8%	Octogenarians MI patients exhibit markedly greater comorbidities and a significantly higher incidence of all-cause death and MACE, even in the DES era.
Minai K *et al*. (2002) [40]	RCT	120/120	82.9	57%	Primary PTCA for very elderly patients with AMI appears to have few beneficial effects on combined events during a 3-year period.

CAD, coronary artery disease; RCT, randomized controlled trial; PCI, percutaneous coronary intervention; STEMI, ST elevation myocardial infarction; CR, complete revascularization; CABG, coronary artery bypass graft; ACS, acute coronary syndrome; MI, myocardial infarction; DES, drug eluting stent; MACE, major adverse cardiac event; GFR, glomerular filtration rate; SPPB, short physical performance battery; NSTEMI, non ST elevation MI.

### 3.2 STEMI in the Elderly: How to Manage Revascularization 

The importance of multivessel disease on prognosis in older MI patients is well established, as it has shown to be an independent predictor of adverse event both in younger [4] and older [2] patients. Therefore, it is of paramount importance to define how to manage this subgroup of patients. Even though the benefit of percutaneous coronary intervention (PCI) versus fibrinolysis in elderly people was debated for years, two randomized trials [41, 42] and one individual patient-meta-analysis [43] favored PCI in terms of death, re-infarction, stroke at 30 day and recurrent ischemia. Furthermore, the current European guidelines [44] on the treatment of STEMI patients do not suggest an upper age limit with respect to reperfusion, especially for PCI. Despite the evidence, up until 2010, in patients aged more than 75, PCI was still underperformed either after STEMI or as PCI rescue after failed fibrinolysis, leading to a higher mortality at 30 days [13, 14, 45]. However, more contemporary data showed how PCI is feasible and without complications in the majority of older patients showing a success rate reaching 99% [15]. By analyzing the Nationwide Inpatient Sample (NIS) registry, Khera *et al*. [14] reported a stable increase in primary PCI for patients >80 years from 9.1% in the year 2000 to 31% in the year 2010. This positive rise in primary PCI is encouraging, and more recent reports registered an almost 80% rate of primary PCI in patients aged over 80 years [38].

Complete revascularization should be considered the gold standard for STEMI patients because it has a positive impact on cardiovascular mortality and repeated revascularization [46]. However, evidence supporting this strategy has been mainly generated in patients with a mean age of around 60 years [46]. Achieving complete revascularization in elderly patients is more challenging, given the major complexity of the lesions and the need for more and longer stents. Studies comparing complete versus culprit-only revascularization in the elderly population are lacking. For instance, no RCTs on this topic have been published yet, and data are mostly built on registries and prospective studies.

The ESTROFA MI +75 registry, is a prospective registry that enrolled 3576 consecutive patients aged more than 75 years who underwent primary angioplasty due to STEMI [16]. A subgroup analysis of 1830 patients with multivessel CAD was conducted to describe the treatment approach and two years outcome. In 847 patients multivessel revascularization was performed and almost two-thirds (566 patients; 67%) of the patients’ complete revascularization (CR) was achieved: not surprisingly, independent predictors of multivessel revascularization were younger age, male sex, previous MI, absence of renal failure and Killip class I-II. Indeed, it was thought that sicker patients could benefit more from a conservative approach [16]. At two years, multivessel PCI was related to a better outcome with an absolute risk reduction of 5% in the combined endpoint of cardiac death and myocardial infarction (HR 0.60, *p* = 0.011), with a greater benefit coming from staged procedure rather than a CR performed during primary PCI. Whereas incomplete revascularization was an independent predictor of adverse event [16]. In addition, the achievement of anatomically defined CR did not influence the 2-year outcome. This seems to suggest that not all the coronary lesions have the same impact on outcome. In addition, functional testing was not performed to assess if those lesions were not only anatomically, but also functionally significant [16].

On the contrary, Rumiz *et al*. [17], found that incomplete revascularization was an independent predictor of major adverse cardiac events (MACE) only in patients younger than 75 years of age. Whereas in older patients, the only independent predictor of mortality was a severe systolic disfunction [17].

In a sub-analysis of the DANAMI-3-PRIMULTI trial focusing on patients >75 years, the authors found a significant interaction between age and treatment assignment (culprit only versus FFR-guided multivessel PCI), with no benefit of a CR approach in elderly patients [18].

Biscaglia *et al*. [19] performed an analysis of four large prospective registries in Europe (mainly from northern Italy) focusing on older MI patients aged 75 years and older. The strategy of revascularization in this population was culprit-only in the majority of patients (65%), confirming that a “real-life” approach to elderly patients with STEMI is conservative [19]. However, also in this analysis, after multivariable adjustment for clinical characteristics, CR was associated with lower mortality with an HR of 0.67 (95% CI 0.50–0.89, *p* = 0.006), primarily driven by the reduction in cardiovascular death. Interestingly, of all the 23 patients that died in the first five days, only one was treated with CR (Table 1).

### 3.3 NSTEMI in the Elderly: How to Manage Revascularization 

Non-ST elevation MI (NSTEMI) is the prevalent clinical presentation in elderly patients with acute coronary syndrome [2]. In addition, patients with NSTEMI have more comorbidities and a poorer short- and long-term prognosis than STEMI patients [47]. The management of these patients is still debated, showing a low rate of PCIs, with medical therapy being the first choice of treatment in the majority of them, excluding high-risk NSTEMI patients, where primary PCI is encouraged.

Furthermore, dedicated RCTs comparing a routine invasive strategy with a selective invasive strategy in elderly patients have shown conflicting results. In a meta-analysis of six trials by Garg *et al*. [48], only 63% of older NSTE-ACS patients underwent revascularization (percutaneous or surgical) in the routine invasive strategy, while only 30% placed at least one stent in the selective invasive strategy group. Performing an invasive approach in every patient reduced the risk of the composite end point of death or MI, primarily driven by a reduction in MI [48].

Similar results were found in the SENIOR-NSTEMI trial [49]. Authors found a 32% lower mortality in the invasive strategy group compared with the non-invasive management: the investigators excluded patients who died in the first three days to limit classification bias [48]. Indeed, assigning patients with an early death to the comparison group could mislead the analysis because some patients could have had invasive management if they did not die.

Most reports from the registries [12, 50] and meta-analyses [51] on surgical approaches in elderly patients are built on data derived from stable patients with chronic CAD, and only a few reports have been done on surgical revascularization after ACS [20, 21, 22]. The latter show more in-hospital mortality in patients undergoing CABG [20, 21, 22] with superiority in terms of incidence of non-fatal MI, revascularization, and death at three years [20, 21]. The authors assert that the long-term survival advantage of surgery is worth the risk of in-hospital death, even in the elderly, and that comorbidities are the factors that most influence the outcomes [20, 21]. In a meta-analysis that included more than 260.000 elderly patients (mean age 75 years), the use of DES was associated with a significant reduction in mortality and subsequent MI [52].

Two retrospective studies [23, 24] on patients with a mean age of 75 and 79 years respectively, and ACS ranging between 54% and 60% compared the revascularization with PCI with either BMS or DES and surgical revascularization. CABG was associated with a significantly lower risk of the combined endpoint of death, MI and revascularization when compared with both BMS or DES (primarily driven by the need for subsequent revascularization) while the all-cause mortality did not differ between DES and CABG [23, 24].

Finally, on the percutaneous revascularization strategy, Harada *et al*. [25] selected 322 elderly patients with multivessel CAD from the SHINANO registry, a prospective, observational, multi-center, all-comer cohort study, where 42% of patients were hospitalized for ACS. Patients were stratified according to complete or incomplete revascularization (ICR). CR drastically reduced the incidence of mid-term MACE, especially ischemic events (ICR 21.1% vs. CR 7.4%, *p *< 0.001). The difference was visible from the acute phase, and the Kaplan-Meier curves kept diverging at one year of follow-up [25]. To reduce the potential selection bias that the non-randomization carries with itself, the authors analyzed data after propensity score matching, and the findings remained consistent. Interestingly, CR was efficient in reducing MACE also after stratification by SYNTAX score and particularly when ACS was the clinical presentation of the patients (Table 1).

### 3.4 Complications after MI in Older Patients

Bleeding is the most frequent non-ischemic complication observed in elderly patients after ACS and is strongly associated with short- and long-term mortality [27, 28, 29]. Age itself is an independent predictor of bleeding in addition to other conditions that are more prevalent in the elderly population, such as chronic kidney disease and atrial fibrillation that require anticoagulation treatment [2]. Several studies report bleeding rate in elderly population ranging between 2 and 5.8% [27, 28], and up to five times higher than in younger patients [38]. In the largest published retrospective series to date, Bromage *et al*. [29] report a 3.43% incidence of bleeding events in octogenarians, defined as access-site bleeding, intra pericardial bleeding, gastrointestinal bleeding, and requirement for blood transfusion. Compared to the younger counterpart, the significant difference in bleeding events was driven by access-site bleeding for the most part, and bleeding complications, as a whole, were independently associated with mortality [29]. Independent predictors of bleeding were age, peripheral vascular disease, female sex, use of intra-aortic balloon pump, administration of Gp IIb/IIIa inhibitors and femoral access, while radial access reduced events [29]. Similarly, other authors reported how bleeding complications could be very low across all age groups if radial access is chosen, strongly favoring this treatment in agreement with recent guidelines [30].

Performing the revascularization of non-culprit lesions via staged procedure requires a new arterial puncture, radial or femoral, with the potential risk of adjunctive bleeding complications.

However, also in elderly patients, considering the revascularization strategy the choice between CR versus culprit only did not impact on the rate of BARC 1 bleeding (2.7% and 2% in complete versus culprit only group, respectively) [11]. Almost the same incidence was found in the study by Harada *et al*. [25].

Finally, older patients undergoing PCI have an increased risk of developing contrast-associated acute kidney injury [53]. It occurs in around 16% of older patients undergoing PCI and it is associated with short-term mortality. Cardarelli *et al*. [31] evaluated the importance of glomerular filtration rate (GFR) in patients with MI that underwent PCI, showing that all complications were more frequent as age increased and renal function declined. In the trial, the authors underline that the calculation of GFR is a better predictor of worst outcome than creatinine alone [31].

### 3.5 Prognosis of Older Patients after ACS

Older patients are more often female, and present more often with hypertension, chronic kidney disease, and reduced ejection fraction, whereas they are less frequently smokers [32, 54]. Also, they have a history of CABG or previous MI more frequent than younger patients [32, 54].

It is not surprising that patients aged 75 years with multivessel CAD, after experiencing an ACS, have a higher rate of in-hospital mortality and higher rate of cardiac death, myocardial infarction and re-admission for heart failure at short- and long-term prognosis [32, 54].

Sakai *et al*. [32] reported an overall in-hospital mortality of 8.4% in older patients, almost doubling the mortality rate of patients <75 years. Caretta *et al*. [38] reported a 30-day mortality of 20%, and one-year mortality of 28%. At a longer follow up of 22 months Rumiz *et al*. [17] showed an incidence of all cause death of 44%, cardiac mortality of 24% and re-admission for HF of 17% [17].

Cardiogenic shock, with a low left ventricle ejection fraction (LVEF), heart failure, hemodynamic instability, higher Killip class, low blood pressure at admission, anterior MI, use of protein IIb/IIIa inhibitors, ventricular arrhythmias, acute stent thrombosis, need of temporary cardiac pacing and low TIMI flow grade after procedure seemed to be the independent factors that most conditioned in-hospital and 30 days mortality despite, what were deemed to be successful PCI [28, 33, 34, 35].

Of particular interest, is the periprocedural MI, which [36] occurred in 4.1% of NSTE-ACS older patients undergoing PCI and increased long-term risks of all-cause and cardiovascular mortality. SYNTAX score, multivessel PCI and total stent length are independent predictors of large periprocedural MI and the occurrence of such complication is associated with poor physical performance at hospital discharge [36].

Finally, in elderly patients, one the most important factors that guide prognosis is frailty [1].

Validated scores to assess prognosis after an ACS, such as GRACE or TIMI scores, are built on baseline characteristics and overlook functional aspects of older patients.

Several scales of frailty have been developed to help the physician in the assessment of physical performance. Campo *et al*. [37] showed that the Short Physical Performance Battery (SPPB) has the greatest incremental value when added to GRACE and TIMI scores in improving the prognostic ability of about 15% in identifying older adults who, despite guideline-based treatment, still have a poor prognosis.

### 3.6 Focus on Nonagenarian Patients

Nonagenarians are a subgroup of patients even more peculiar than their younger counterpart (Table 2, Ref. [55, 56, 57, 58]). Rigattieri *et al*. [55] reported a higher prevalence of the female gender, and a low percentage of common risk factors, which may result from a selection bias as these characteristics allow the population to achieve such an advanced age. This population is particularly underrepresented in RCTs. The available evidence comes from a few small observational trials [56, 57, 58]. In nonagenarians, mortality appears to be significantly higher when angioplasty is performed in the setting of unstable CAD. Moreno *et al*. [56] studied a population mostly presenting with ACS and undergoing PCI. Intrahospital mortality was 19% despite the high angiographic success rate (92%), defined as obtainment of TIMI 2–3 flow after the procedure. Survival rate was 69% at one month, and 65% at one year. In another retrospective analysis of nonagenarians undergoing PCI from Teplitsky *et al*. [57], immediate procedural success rate, was high (92%). However, mortality in the ACS setting was significant, reaching 23% at six months. In nonagenarians STEMI patients [55] referred for primary PCI, in-hospital mortality reached 18% and procedural success achieved in 89%. Interestingly, the pain-to-balloon time was consistently long (6.25 hours). The delayed presentation to the emergency room may depend on the difficulty of understanding and interpreting symptoms in the very elderly. No data are available about revascularization strategies (complete versus culprit only) in nonagenarians MI patients.

**Table 2.** **Studies included in the systematic review on nonagenarians**.

References	Study Type	Patients	Outcome
Revascularization in nonagenarians with acute coronary syndrome			
Rigattieri S *et al*. 2013 [55]	Retrospective single center cohort	27	Primary PCI is feasible and effective in nonagenarian patients with STEMI. Most adverse events are confined to the early phase (within 30 days from admission). Major bleeding seems not to be an issue and should not discourage the administration of guideline- recommended antithrombotic therapies.
Moreno R *et al*. 2004 [56]	Retrospective study	29	PCI achieves a successful angiographic result in most cases. In-hospital mortality occurred only in patients in cardiogenic shock or in those with primary angioplasty as PCI.
Teplitsky I *et al*. 2007 [57]	Retrospective analysis	65	Prognosis among nonagenarians undergoing emergent PCI is acceptable.
LeBude B *et al*. 2012 [58]	Retrospective cohort	44	Diagnostic and interventional cardiac catheterization is safely performed in a select group of nonagenarian patients with therapeutic benefit and 80% survival at 12 months.

PCI, percutaneous coronary intervention; STEMI, ST elevation myocardial infarction.

## 4. Conclusions

**Fig. 2.**
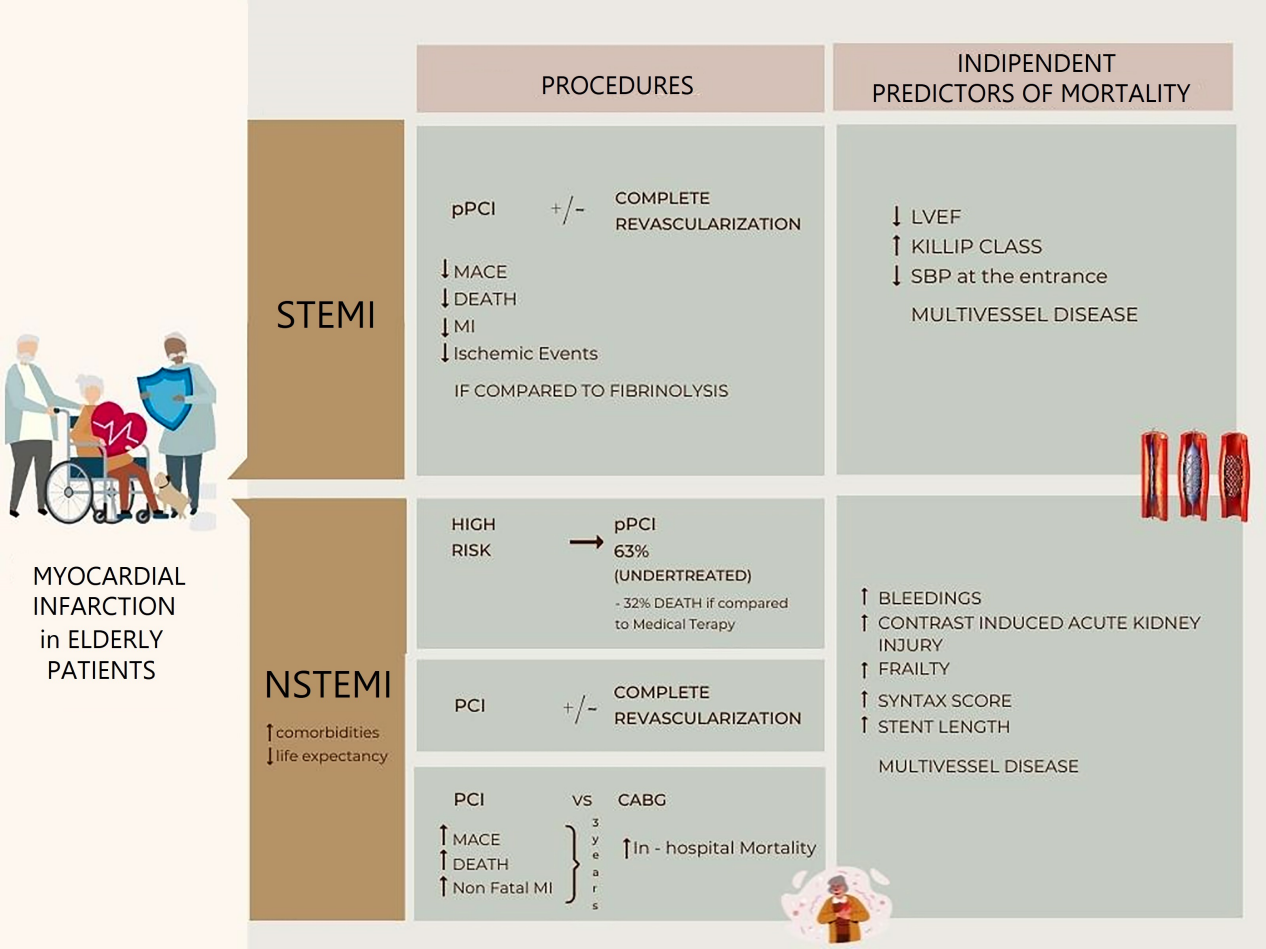
**Elderly patients with myocardial infarction and multivessel disease**. This is a frail population who is under-represented in randomized clinical trials and is mainly treated without specific guidelines or indications. Elderly patients usually have a multivessel disease with chronic and calcified lesions. It is a population affected by comorbidities: chronic kidney disease, previous cardiac revascularization, and hypertension. After revascularization, older MI patients have a worse prognosis compared to their younger counterparts showing increased in-hospital mortality, cardiac death, myocardial infarction, and heart failure. In this population, the procedural bleeding rate after percutaneous coronary intervention is usually higher. LVEF, left ventricle ejection fraction; CABG, coronary artery bypass graft; MI, myocardial infarction; pPCI, primary percutaneous coronary intervention; SBP, Systolic Blood Pressure; PCI, percutaneous coronary intervention; MACE, major adverse cardiovascular events.

This systematic review shows that there is a lack of RCTs to guide the revascularization strategy in older STEMI or NSTEMI MI patients (Fig. 2). New results are expected from two ongoing trials, namely the FIRE trial [59] (NCT03772743) and the SENIOR-RITA trial [49, 60] (NCT03052036). The FIRE trial is a prospective, randomized, international, multicenter, open-label study, enrolling 1400 older MI patients (either STEMI or NSTEMI, aged 75 years and older), with multivessel CAD. Patients will be randomized to culprit-only treatment or to physiology-guided CR. The primary endpoint will be the patient-oriented composite end point of all-cause death, any MI, any stroke, and any revascularization at 1 year. The key secondary endpoint will be the composite of cardiovascular death and MI [59].

The SENIOR-RITA trial (PMID: 32861307) will enroll 2300 patients with NSTEMI aged 75 years or older and its completion date is expected to be 2029. The trial will analyze whether an invasive management strategy compared with a non-invasive one reduces time of cardiovascular death or non-fatal myocardial infarction in that population [60].

Results of these two large trials will provided the much needed answers to the questions about the management of older patients. Until the publication of the results of these two trials, the literature data suggest treating patients aged 75 years and older as young patients involved in the current trials, i.e., favoring complete revascularization in the case of multivessel CAD, primary angioplasty in STEMI rather than fibrinolysis, and drug-eluting stents. We should pay close attention to frailty and the physical performance status both influencing prognoses, as well as to factors favoring bleeding and chronic kidney disease. Therefore, cardiologists should prefer a complete revascularization over a culprit only one, also in the elderly. However, at this stage of research and knowledge on the topic in the case of multi-comorbid subjects, the heart team should make decisions on the best revascularization strategy based on individual cases after a frailty assessment.
